# From Soil to Brain: Olive Oil Attributes, Consumer Choices, Intermittent Fasting, and Their Impact on Health

**DOI:** 10.3390/nu17111905

**Published:** 2025-06-01

**Authors:** Ion-Bogdan Dumitrescu, Cristina Manuela Drăgoi, Alina Crenguța Nicolae

**Affiliations:** 1 Department of Physics and Informatics, Faculty of Pharmacy, Carol Davila University of Medicine and Pharmacy, 6, Traian Vuia St., 020956 Bucharest, Romania; ion.dumitrescu@umfcd.ro; 2 Department of Biochemistry, Faculty of Pharmacy, Carol Davila University of Medicine and Pharmacy, 6, Traian Vuia St., 020956 Bucharest, Romania; alina.nicolae@umfcd.ro

**Keywords:** olive oil, extra-virgin olive oil, EVOO, polyphenols, fasting

## Abstract

Olive oil (OO) has longstanding significance in human history, particularly in the Mediterranean region, where it has been a cornerstone of diet, economy, and culture. This history adds to modern evidence-based knowledge. **Background**: The Mediterranean diet (MD), rich in plant-based foods and OO, has been extensively associated with improved cardiometabolic and cognitive health. Recent interest has emerged in understanding how intermittent fasting protocols may enhance these effects. Still, the quality of OO does not only lie in the extraction process; it is also dependent on the tree variety, the soil, and the agricultural practices, ending with the way in which the finished product is stored and consumed. **Objectives**: This review explores the synergistic potential between OO consumption and intermittent fasting, focusing on their combined impact on metabolic health, oxidative stress, and inflammatory pathways. **Methods**: A literature search was conducted using multiple databases to identify studies addressing the health effects of OO, fasting, and the MD. Both human and relevant preclinical studies were considered, with emphasis on those evaluating inflammatory markers, lipid metabolism, insulin sensitivity, and neuroprotective mechanisms. **Results**: Evidence suggests that the bioactive compounds in EVOO may potentiate the benefits of fasting by enhancing antioxidant capacity, reducing postprandial inflammation, and modulating gene expression related to cellular metabolism. Combined, these factors may support improved insulin sensitivity, reduced oxidative damage, and delayed onset of age-related diseases. **Conclusions**: Understanding the integrative role of OO and fasting within the MD framework could offer valuable insights for nutritional strategies aimed at preventing metabolic syndrome, type 2 diabetes, and neurodegeneration. These findings also support the need for future clinical trials exploring the timing, dosage, and dietary context in which these interventions are most effective.

## 1. Introduction

“*The Mediterranean peoples began to emerge from barbarism when they learned cultivating the olive tree and the grape vine*”, the Athenian historian Thucydides was reported to have said in the fifth century BCE [1,2], and indeed, archeological findings indicate that extensive olive cultivation and olive oil (OO) trade spread from the coastal areas of the Levant since the late Neolithic and early Bronze Age (3000 BCE) [3,4]. Now only a fraction of the world’s olive oil is still produced in the Levant, while the main producers have moved to the west of the Mediterranean: for the 2018–2023 interval, 41% of the global production was in Spain, 8.8% in Italy, and 8.3% in Greece [5]. Since the Roman period, olive oil has been the main fat consumed in the Middle East [4], and this is still true today for the contemporary Mediterranean diet (MD) [6], but reliable information is scarce on the actual amount consumed per capita. United Nations data estimates the annual per capita olive oil consumption in 2021 at 14 kg in Greece, 12 kg in Spain, and 10 kg in Italy [7]. In a recent Lebanese study, a third of households consumed more than 30 L per year, with more than one quarter of the respondents consuming approximately 2 teaspoons of olive oil each day [8]. The methodology of the current review is detailed in Appendix A.

## 2. The Context of the Mediterranean Diet and Fasting

Epidemiological research on the MD started in the 1950s, and since then, studies confirmed that this diet, in which extra-virgin olive oil (EVOO) represents around 85% of the total fat intake and up to one third of the total caloric intake, decreased the incidence of hypercholesterolemia, atherosclerosis, diabetes, obesity, hypertension, and neurodegenerative diseases and showed a significant drop in total mortality and in cancer risk, with additional antimicrobial and anti-inflammatory activities [1,9,10]. The health benefits of the OO-rich MD are shown by studies and prevail over those of low-fat diet models [6]. On the other hand, diets in which EVOO intake exceeds 35% of the total caloric intake are not beneficial [10].

When evaluating the health benefits of the MD, it is important to consider not only the composition of the diet itself but also fasting practices, as the Mediterranean region is home to diverse religious traditions that incorporate various forms of fasting, ranging from intermittent abstention from certain food groups to prolonged periods of caloric restriction, which may play a significant role in the overall health outcomes traditionally attributed to the MD [11].

Dietary interventions like intermittent fasting and caloric restriction significantly influence gut microbiota, but findings on their effects remain inconsistent due to study heterogeneity [12,13]. Animal studies suggest that microbiota composition adapts to feeding modifications, but results vary across bacterial strains and regimens, and human studies show shifts in bacterial diversity and activity depending on age, ethnicity, sex, and health status, with some changes being transient [12]. Some fasting regimens involving sirtuins (SIRT)-activating compounds, as is oleic acid—the major component of OO—show potential benefits, yet conclusive patterns are not evident [12,14].

Fasting, including intermittent fasting and time-restricted feeding, has been shown to improve insulin sensitivity, promote fat oxidation, and reduce markers of oxidative stress and inflammation [15]. Fasting also activates autophagy and modulates metabolic signaling pathways such as AMP-activated protein kinase (AMPK) and mammalian target of rapamycin (mTOR), contributing to cellular resilience and longevity [16].

Fasting and the MD are two distinct yet complementary nutritional strategies that have demonstrated robust benefits in metabolic, cardiovascular, and inflammatory conditions. When combined, particularly with EVOO as a shared core component, these approaches may exert synergistic effects that enhance health span and reduce disease risk [17,18].

Integrating fasting with a Mediterranean-style dietary framework, particularly in the refeeding phases or in modified fasting protocols, enhances nutrient density while maintaining metabolic flexibility.

EVOO plays a central role in this integration by

- Supporting satiety during fasting periods [19];
- Providing anti-inflammatory effects that complement fasting-induced reductions in pro-inflammatory cytokines [14];
- Minimally disrupting ketogenesis and glycemic control when consumed in small quantities during modified fasts [20];
- Offering gut and cardiovascular protection during refeeding phases [21].

Furthermore, studies such as the PREDIMED trial have shown that higher adherence to an MD supplemented with EVOO leads to improved lipid profiles, lower blood pressure, and decreased risk of major cardiovascular events [22].

The combination of fasting and an MD enriched with EVOO represents a powerful, evidence-based strategy for improving cardiometabolic health, enhancing cellular function, and potentially extending health span [17].

## 3. Chrononutrition, EVOO Intake, and Health, from a Biochemical Perspective

Recent research in chrononutrition, a field that investigates the temporal organization of nutrient intake in relation to endogenous circadian rhythms, has begun to elucidate how the timing of food consumption modulates metabolic and physiological outcomes [23,24,25,26,27]. The synchronization between nutrient intake and circadian biology is increasingly recognized as a determinant of cardiometabolic health [28,29]. Within this framework, the incorporation of OO, particularly EVOO, into the diet warrants attention due to its rich profile of bioactive compounds and functional lipids. EVOO, a principal source of monounsaturated fatty acids in the Mediterranean diet, is predominantly composed of oleic acid and a diverse array of phenolic compounds including hydroxytyrosol, oleuropein, and oleocanthal [30]. These constituents were associated with anti-inflammatory, antioxidant, and cardioprotective effects, which may interact with circadian-controlled metabolic pathways [30].

Scientific evidence suggests that the metabolic processing of dietary lipids, including absorption, lipoprotein metabolism, and postprandial lipid clearance, is under circadian regulation [31]. Insulin sensitivity and lipolytic activity exhibit diurnal variation, with peak metabolic efficiency observed in the early part of the day. Accordingly, the consumption of EVOO during morning or early afternoon meals may enhance lipid utilization and reduce postprandial lipemia, a recognized cardiovascular risk factor [32].

Moreover, the bioavailability and systemic efficacy of EVOO polyphenols appear to be modulated by the time of ingestion. Enzymatic activity within the gastrointestinal tract, intestinal permeability, and hepatic xenobiotic metabolism—all crucial for polyphenol absorption and transformation—are subject to circadian modulation [33]. Therefore, aligning EVOO intake with periods of optimal gastrointestinal and hepatic activity may potentiate its systemic antioxidant and anti-inflammatory effects [33].

Oleocanthal, a phenolic compound unique to EVOO, exerts anti-inflammatory activity through cyclooxygenase (COX) inhibition, mimicking the pharmacological action of nonsteroidal anti-inflammatory drugs [34]. Given the circadian rhythm in pro-inflammatory cytokine secretion and immune cell activation, timed consumption of EVOO may act synergistically with these oscillations to modulate inflammatory responses [34].

The elderly population, who often exhibit disrupted circadian rhythms and increased oxidative stress, may particularly benefit from structured EVOO consumption during the active phase of the day (i.e., morning to early afternoon). This strategy may support metabolic homeostasis and attenuate age-related pathologies including cardiovascular disease and neurodegenerative conditions [35].

In the context of the Mediterranean dietary pattern, which emphasizes early meal timing, plant-based foods, and liberal use of EVOO, there is inherent compatibility with chrononutritional principles. Epidemiological and interventional studies demonstrated that the MD, when consumed in synchrony with circadian rhythms, confers superior metabolic and cardiovascular outcomes compared to isocaloric diets with suboptimal temporal patterns [36,37].

## 4. Nutritional Strategies in the Context of Therapeutic Modulation

Fasting, defined as a voluntary abstention from caloric intake for varying durations, has gained increasing attention for its potential therapeutic effects on metabolic health, inflammation, and cellular repair mechanisms. Various forms of fasting, including intermittent fasting, time-restricted feeding, and prolonged fasting, were shown to modulate insulin sensitivity, enhance autophagy, and promote fat oxidation [15,21]. Within this context, the inclusion or strategic use of specific nutrients such as EVOO has emerged as a topic of scientific interest [30].

Olive oil, particularly EVOO, is characterized by a high content of monounsaturated fatty acids, predominantly oleic acid, and a wide range of bioactive compounds including polyphenols, squalene, and tocopherols that possess well-documented anti-inflammatory, antioxidant, and cardioprotective properties [30,38].

In modified fasting protocols, where minimal caloric intake is permitted, small quantities of OO may be used without substantially disrupting the physiological fasting state. Due to its low glycemic impact and negligible effect on insulin secretion, OO does not significantly interfere with key metabolic pathways associated with fasting, such as ketogenesis and lipolysis [20]. Moreover, its high satiety index may help mitigate hunger and improve adherence to fasting regimens [19].

The bioactive constituents of EVOO are also implicated in supporting mitochondrial function, reducing oxidative stress, and modulating inflammatory cytokine expression, all of which align with the mechanistic goals of fasting. Additionally, during the refeeding period post-fast, the incorporation of OO can support digestive reactivation in a gentle manner, enhance nutrient absorption, and promote bile production, aiding in the metabolism of dietary fats [30].

From a clinical nutrition standpoint, the synergistic application of fasting and EVOO may offer a complementary approach in the management of metabolic disorders, including type 2 diabetes, obesity, and cardiovascular disease. However, further randomized controlled trials are necessary to delineate optimal dosages, timing, and long-term effects of EVOO consumption within various fasting protocols [39].

Current evidence suggests that EVOO, due to its unique lipid profile and bioactive components, may enhance the tolerability and efficacy of fasting interventions while preserving key metabolic benefits [30].

When combined, the fasting regimen and OO consumption may exert synergistic effects in addressing the pathophysiological components of metabolic syndrome. Fasting improves metabolic flexibility and fat utilization, while EVOO provides essential fatty acids and bioactive compounds that support cardiovascular and metabolic health without disrupting the metabolic state induced by fasting. Additionally, the inclusion of EVOO during refeeding or in modified fasting protocols may enhance satiety, maintain lipid homeostasis, and attenuate postprandial glycemic spikes [40].

Fasting, particularly in its prolonged or intermittent forms, leads to periods where the stomach remains empty for extended durations. In this state, gastric mucosal protection is reduced due to lower prostaglandin levels and less mucus secretion, thereby increasing susceptibility to nonsteroidal anti-inflammatory drug (NSAID)-induced mucosal injury. Consequently, the use of ibuprofen during fasting is generally discouraged unless accompanied by protective strategies [40].

One such strategy may involve EVOO, which possesses gastroprotective, anti-inflammatory, and antioxidant properties. EVOO is rich in monounsaturated fatty acids (primarily oleic acid) and polyphenolic compounds, some of which exhibit ibuprofen-like COX-inhibitory activity without the same gastrointestinal toxicity. Moreover, EVOO enhances mucosal defense by increasing prostaglandin E2 synthesis and reducing oxidative damage to the gastric lining [34].

While OO should not be considered a replacement for pharmaceutical gastroprotectants in high-risk NSAID users, moderate intake during or prior to NSAID administration, particularly in fasting states, may provide a natural protective buffer to reduce mucosal damage. Additionally, OO’s anti-inflammatory effects may synergize with or reduce the required dose of ibuprofen in certain contexts, though clinical data on this interaction remain limited [41].

Fasting increases the risk of ibuprofen-induced gastrointestinal irritation, while OO may offer a protective and complementary role. Co-ingestion of OO or avoiding NSAID use on an empty stomach may help mitigate adverse effects. However, more research is needed to clarify the optimal timing, dosage, and safety of combining these elements in clinical or self-care settings [42].

## 5. The Olive Fruit and the Types of Olive Oil

**Figure 1:**
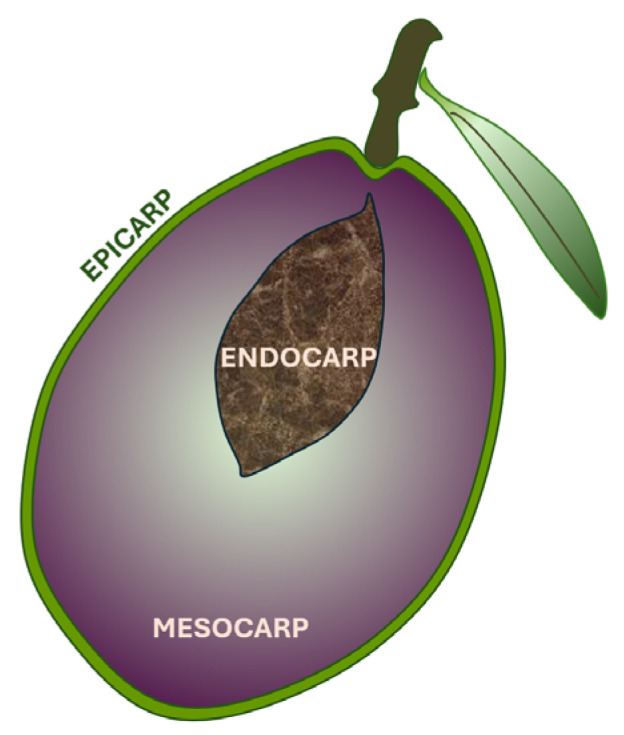
The structure of the fresh olive drupe: the epicarp represents the skin, the mesocarp represents the pulp or the flesh, and the endocarp represents the stone or the pit, containing the kernel or the seed.

The water content of fresh olives ranges between 50 and 70% and oil between 20 and 30%, while carbohydrates represent less than 19%, cellulose 6%, and protein and minerals 1.5% each [4,43]. The structure of the olive fruit is detailed in Figure 1.

Olive trees follow a biennial bearing cycle, producing heavy crops (ON) and light crops (OFF) in alternating years. During ON years, fruit development limits shoot growth and reduces flowering for the next season. ON years result in smaller fruits with a lower flesh-to-stone ratio, delayed ripening, and less oil. However, the overall oil production per tree remains higher in ON years [43].

Oil represents 14–30% of the mesocarp and only 1–1.5% of the endocarp [10,43]. The oil from the mesocarp agglomerates in vacuoles, while that in the endocarp is cytoplasmatic [44].

**Figure 2:**
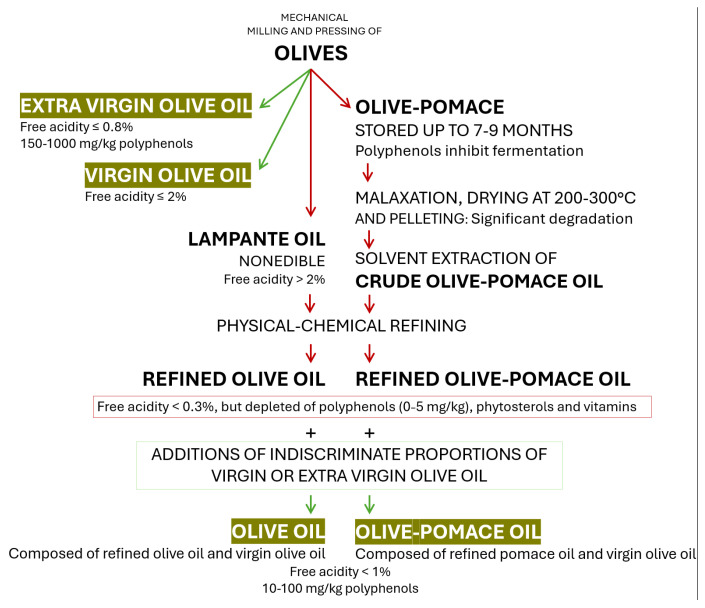
An overview of OO extraction methods shows how the ratio of polyphenols and other beneficial components of OO is heavily dependent on the technological process of extraction. The green highlight designates the four commercially available OO types, with legal labels and free acidities set by EU regulation [47,49,50]. Lampante oil is a very low-quality oil, either obtained from low-quality or old olive fruit or improperly processed, which makes it inedible without further refining [47].

This means that physical pressing could be sufficient to extract almost all the vacuolar oil from the mesocarp, while the cytoplasmatic oil from the kernel, even if crushed, is much more difficult to extract and remains in the pomace [44]. For an overview of OO extraction processes, see Figure 2.

Even so, malaxation is an essential process, as it enhances oil droplet coalescence and the separation of phases, as well as promoting the formation of volatile compounds, leading to a highly nutritional and flavorful oil, as it allows polyphenols and other minor constituents to disperse in the oil physically or due to enzymatic activity [10,45,46].

Oil separation is carried out by one of three methods: mechanical pressing, percolation, or centrifugation [45]. The remaining pomace can be stored for months and is further processed to lose most of the moisture, and then the remaining oil is extracted using solvents like hexane [45,47].

The use of n-hexane as an oil extraction solvent is somewhat justified in the case of sunflower seeds, for example, where it can boost the yield from 25 to 40% when compared to using pressure alone, but in the case of olive pomace, only a small remaining percentage of residual oil is extracted, and following this process of making the crude olive pomace oil edible, polyphenols, phytosterols, vitamins, and other bioactive molecules are lost [45,48,49]. Traces of polyphenols can still be found in commercial regular olive or olive pomace oil because of the legal obligation to mix refined OOs and refined olive pomace oils with various proportions of EVOO or VOO [47].

In fresh olives, the phenolic contents could amount to a striking 20–30 g/kg in the mesocarp, but because of their polar and thus more hydrophilic nature, only a minute fraction ends up in the EVOO after pressing and filtering—on average only 0.5 g/kg (0.05%)—with the rest persisting in the pomace (~45%) and the majority being lost in wastewater (~53%) or degraded during refining processes [45,49,51,52]. Some of the phenolic constituents become oil-soluble only after they are enzymatically hydrolyzed and form aldehydic aglycones [45,46].

## 6. EVOO Constituents and Their Health Attributes

### 6.1. Legal and Regulatory Considerations

It is important to acknowledge first that each effect is dependent on the dose and also on the frequency of administration, so this is why all the health claims allowed commercially at the European level by the EU Commission Regulation 432/2012 concerning different constituents of EVOO must specify the minimum or maximum daily intake needed for each specific constituent to have the specific beneficial health outcome listed [53]. For example, the claim “*Olive oil polyphenols contribute to the protection of blood lipids from oxidative stress*” may be used only if the oil has a minimum of 0.025% content in hydroxytyrosol and derivatives and only if the consumer is also instructed to consume a daily minimum of 20 g of oil [53]. The average value identified in the literature for polyphenol content in EVOO is 0.05% [49].

The World Health Organization (WHO) updated on 17 July 2023 its guidance on total fat, saturated fat, and trans-fat based on the latest scientific evidence. Table 1 presents a summary of the European Food Safety Authority (EFSA) and WHO guidelines on some EVOO constituents and health claims.

The WHO emphasizes the significance of both the quantity and quality of dietary fat for maintaining optimal health. Although the WHO’s “Healthy Diet” guidelines do not specify OO *per se*, they underline that adults should restrict their total fat intake to a maximum of 30% of their total energy consumption. For individuals aged two years and older, fat intake should predominantly consist of unsaturated fatty acids. Saturated fatty acids should contribute no more than 10% of total energy intake, while trans-fatty acids (TFAs), whether derived from industrial processing or ruminant animal sources, should be limited to less than 1% of total energy intake [54]. This leaves room for incorporating monounsaturated fats, such as those found in OO, which are well-recognized for their beneficial effects on lipid profiles and overall cardiovascular health.

To promote healthier dietary patterns, saturated and trans-fatty acids can be substituted with alternative macronutrients, such as polyunsaturated fatty acids, monounsaturated fatty acids from plant-based sources, or carbohydrates rich in naturally occurring dietary fiber, including whole grains, vegetables, fruits, and legumes. Saturated fatty acids are predominantly found in fatty meats, dairy products, and solid fats and oils, such as butter, ghee, lard, palm oil, and coconut oil. Meanwhile, trans-fatty acids are commonly present in processed foods, including baked and fried products, pre-packaged snacks, and animal-derived foods from ruminants such as cows and sheep [55].

### 6.2. Age-Specific Considerations for Lipids Intake

For children, the focus is on ensuring a balanced intake of fats that supports growth and cognitive development. While specific EVOO recommendations for children are less commonly delineated in major guidelines, incorporating EVOO as a primary source of dietary fat within the framework of a balanced diet can help provide essential fatty acids and antioxidants. National dietary guidelines (such as those from Mediterranean countries) often suggest that healthy fats, including EVOO, should make up a substantial part of the daily fat intake of children, adjusted proportionally to their lower energy requirements [55,56].

In adult populations, many MD guidelines recommend daily consumption of around 25–30 mL (approximately 2–3 tablespoons) of EVOO as a key component of a heart-healthy diet. This amount is consistent with findings from the PREDIMED study, which showed a reduction in cardiovascular risk with increased OO consumption. Such intake supports the maintenance of healthy lipid profiles and provides anti-inflammatory benefits [22].

For elderly individuals, nutritional needs are similar to those of adults but with increased emphasis on maintaining cardiovascular health and preventing age-related oxidative stress. The inclusion of EVOO is particularly beneficial for this group [55,57,58].

### 6.3. Olive Oil in the Context of Mediterranean Diet

The high content of monounsaturated fatty acids and antioxidants underpins many of the diet’s beneficial effects, including reduced inflammation, improved endothelial function, and enhanced metabolic control [27,55].

Recent studies continue to reinforce the connection between EVOO consumption and lower risks of cardiovascular diseases, type 2 diabetes, and even certain types of malignancies [59]. Moreover, the MD’s synergy, where EVOO works in concert with other nutrient-dense foods, creates a holistic approach to preventing chronic diseases and promoting longevity [60,61].

In summary, current recommendations from the WHO and EFSA support the inclusion of EVOO as a beneficial fat source within a balanced diet. While specific quantitative guidelines may vary by age group, the overall evidence supports daily intakes of 20–30 mL for adults and the elderly, with proportionally adjusted amounts for children. The MD remains a prime example of how EVOO can contribute to health through its anti-inflammatory, antioxidant, and metabolic-modulating effects [62,63].

### 6.4. EVOO Composition and Organoleptic Attributes

EVOO is one of the best sources of nutraceuticals, and although most of the published studies are in vitro, the results are promising for a wide range of chronic and degenerative pathologies [6].

EVOO and VOO composition varies depending on the cultivar genotypes, soil properties, climate, sanitary and agronomic conditions, ripeness stage of olives, harvesting method and degree of fruit damage, extraction method, and, last but not least, packing materials, storage, and cooking conditions [43,47,49,64]. The average composition of EVOO is presented in Table 2.

The hue, scent, flavor, taste, and aftertaste of EVOO are influenced by its minor fraction [10]. EVOO’s organoleptic properties, influenced by polyphenols, contribute to its sensory qualities like bitterness and throat irritation [8], which does not make them easily accepted by most consumers [6]. The bitterness or the astringency notes are influenced by some of its minor components, such as secoiridoids like oleuropein and ligstrozide [6,49]. Ligstrozide and oleocanthal are responsible for the irritant burning throat sensation that is characteristic of high-quality EVOOs [6].

During the organoleptic assessment of EVOOs and VOOs, a highly trained panel of tasters identifies sensory attributes (fruitiness, bitterness, pungency) or defects (such as musty, winey, or rancid aromas) [43].

### 6.5. Health Benefits of EVOO

**Figure 3:**
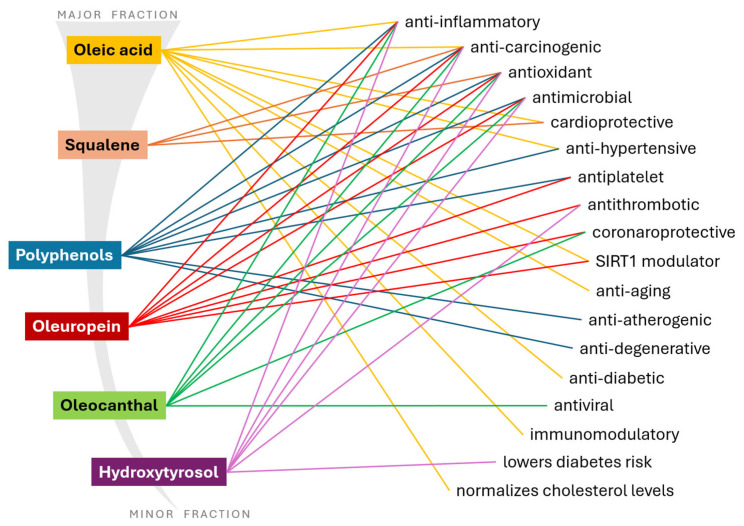
Protective effects of olive oil’s major and minor constituents on health and disease processes.

EVOO has received considerable attention for its health-promoting properties, derived primarily from its lipidic composition, characterized by a high content of monounsaturated fatty acids (MUFAs)—especially oleic acid—and its rich spectrum of minor bioactive compounds such as polyphenols [1,6,61]. These are summarized in Figure 3.

#### 6.5.1. Cardiovascular Health

The cardioprotective effects of EVOO are closely linked to its lipid profile. EVOO contains approximately 70–80% oleic acid, a MUFA well-recognized for its beneficial cardiovascular properties. Moreover, EVOO has an optimal omega-6 to omega-3 fatty acid ratio (ω6:ω3 between 5:1 and 10:1), contrasting sharply with the typical Western diet, which usually has a ratio around 16:1, associated with pro-inflammatory and pro-atherogenic effects. Additionally, EVOO is low in saturated fatty acids (SFAs), further supporting its cardiovascular protective profile. Regular intake of EVOO results in decreased plasma levels of LDL and VLDL cholesterol alongside an occasional increase in HDL cholesterol, collectively reducing risks associated with atherosclerosis and coronary heart disease. The Food and Drug Administration (FDA) acknowledges that a daily intake of approximately 23 g of EVOO can reduce coronary heart disease risk provided it substitutes for an equivalent amount of saturated fats. Similarly, EFSA highlights the cardiovascular benefits of replacing dietary saturated fats with unsaturated fats, as per Regulation EU 432/2012, to maintain normal plasma cholesterol levels [10,18,43,53,54,55,66].

#### 6.5.2. Anti-Inflammatory Effects

**Figure 4:**
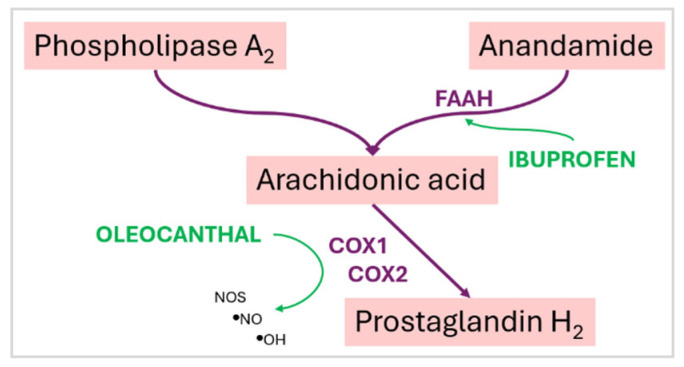
Involvement of ibuprofen and oleocanthal in the prostaglandin pathway. FAAH—fatty acid amide hydrolase. COX1 and COX2—cyclooxygenase 1 and 2. Adapted from [67,68,69].

EVOO exerts potent anti-inflammatory activities through both its fatty acids and polyphenolic constituents. Oleic acid helps modulate inflammation by gradually replacing pro-inflammatory fatty acids such as linoleic and arachidonic acids in cell membranes. This substitution influences key inflammatory signaling pathways, notably the nuclear factor kappa-light-chain-enhancer of activated B cells (NF-κB) pathway, reducing pro-inflammatory cytokine expression. The polyphenolic compound oleocanthal, found in EVOO, inhibits COX-1 and COX-2 enzymes in a manner analogous to NSAIDs like ibuprofen, offering dose-dependent anti-inflammatory benefits. COX-1 and COX-2 enzymes are critical components of the prostaglandin synthesis pathway, as shown in Figure 4 [67,68].

Furthermore, polyphenols such as hydroxytyrosol and oleuropein suppress other inflammatory mediators, reinforcing the preventive role of EVOO against chronic inflammatory states linked to various degenerative diseases [10,14,67,70,71,72].

#### 6.5.3. Antioxidant Activities

The antioxidant capacity of EVOO is significantly attributed to its polyphenolic content, including compounds such as hydroxytyrosol, tyrosol, oleuropein, and oleocanthal [61,73,74,75]. These antioxidants actively scavenge reactive oxygen species (ROS), limiting oxidative damage to cellular components such as proteins, lipids, and DNA. EVOO polyphenols enhance endogenous antioxidant enzyme activities, including superoxide dismutase, catalase, and glutathione peroxidase, further fortifying cellular defense mechanisms [76,77,78]. EFSA recognizes the antioxidant properties of hydroxytyrosol and related polyphenols, recommending an intake of at least 5 mg/day through EVOO consumption to achieve significant antioxidative protection, notably the inhibition of LDL oxidation, a key factor in atherogenesis [39,70,79,80,81,82,83].

#### 6.5.4. Neuroprotective Potential

**Figure 5:**
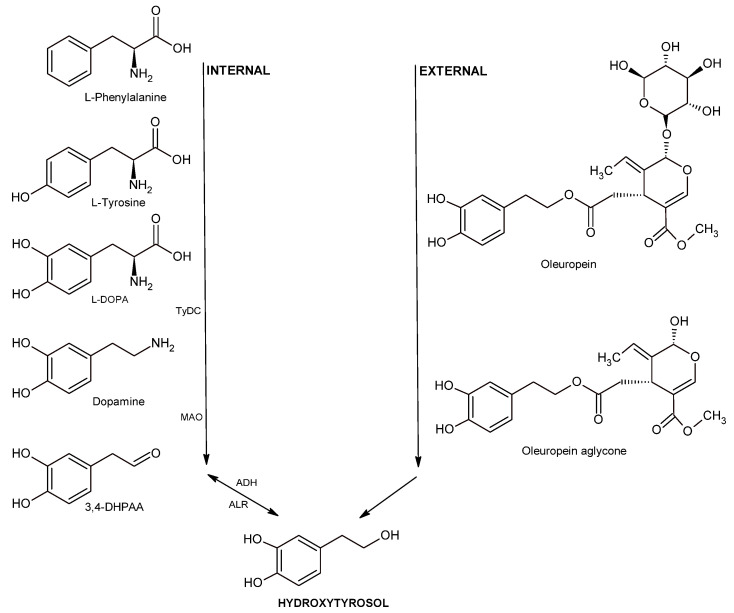
Hydroxytyrosol may be generated endogenously through dopamine metabolism or acquired exogenously. TyDc—tyrosine/DOPA decarboxylase, MAO—monoamine oxidase, ADH—alcohol dehydrogenase, ALR—aldehyde reductase [91,92,93].

Emerging evidence highlights the potential neuroprotective benefits of EVOO, primarily attributable to its polyphenolic components, notably oleocanthal, oleuropein, and hydroxytyrosol. These polyphenols demonstrate the ability to traverse the blood–brain barrier, exerting direct antioxidant and anti-inflammatory actions within neural tissues [84,85,86,87,88]. Through the activation of intrinsic protective mechanisms such as the nuclear factor erythroid 2-related factor 2 (Nrf2) pathway, EVOO phenolics bolster neuronal resilience to oxidative stress and inflammation. Specifically, oleocanthal promotes cerebral clearance of amyloid-beta (Aβ), suggesting therapeutic potential in neurodegenerative conditions such as Alzheimer’s and Parkinson’s diseases. Moreover, EVOO polyphenols attenuate neuroinflammation, a critical factor in cognitive decline and neurological disorders. Notably, hydroxytyrosol has intriguing biochemical connections to neurotransmitter metabolism, particularly involving dopamine synthesis pathways, as depicted in Figure 5 [89,90].

The dopamine biosynthetic pathway commences with L-phenylalanine, which is converted into L-tyrosine. This L-tyrosine is then transformed into L-3,4-dihydroxyphenylalanine (L-DOPA), the immediate precursor to dopamine. Next, tyrosine/DOPA decarboxylase catalyzes the conversion of L-DOPA into dopamine, which is subsequently metabolized into 3,4-DHPAA by monoamine oxidase (MAO) [89]. The final step of this metabolic pathway involves the production of hydroxytyrosol via a reversible reaction catalyzed by alcohol dehydrogenase [91,94].

Conversely, the external pathway for hydroxytyrosol formation in olives during maturation is comparatively simpler. In this process, a β-glycosidase converts oleuropein into its aglycone form, which then undergoes hydrolysis to yield elenolic acid and hydroxytyrosol [94].

#### 6.5.5. Metabolic Regulation and Longevity

The metabolic regulatory and potential longevity benefits associated with EVOO are predominantly linked to its MUFA content, especially oleic acid, as well as to its bioactive polyphenols, such as oleuropein and hydroxytyrosol. EVOO polyphenols influence key metabolic pathways by activating SIRT1, an NAD⁺-dependent enzyme pivotal in cellular metabolism, stress resistance, and aging processes. Activation of SIRT1 by EVOO components has been associated with improved mitochondrial function, increased insulin sensitivity, and reduced inflammation, thereby promoting metabolic health. These mechanisms collectively enhance cellular resilience, potentially delaying the onset of age-related metabolic disorders, including metabolic syndrome and diabetes, and possibly contributing to increased longevity [12,14,76,95,96].

#### 6.5.6. Modulating Membrane Potential and Fluidity

The bioactive components of EVOO, particularly oleic acid and polyphenols such as hydroxytyrosol and oleuropein, play a crucial role in modulating membrane potential and fluidity. Oleic acid integrates into phospholipid bilayers, enhancing membrane fluidity and elasticity, which supports optimal functioning of membrane-bound proteins, ion channels, and receptors. This modulation of membrane dynamics influences a range of physiological processes including cellular signaling, neurotransmission, nutrient transport, and energy metabolism. Polyphenols further contribute by stabilizing membrane structures and protecting them from oxidative damage, thereby preserving the biophysical properties necessary for cellular homeostasis and metabolic regulation [97,98,99]. Furthermore, these compounds can prevent apoptosis, including cell death induced by H_2_O_2_, a key property given the critical role of cell death in neurodegenerative processes’ development. Polyphenols confer cytoprotection by hyperpolarizing the basal mitochondrial membrane potential and by reducing the activity of neuronal Na⁺/K⁺ ATPase [82,97,100].

#### 6.5.7. Anticancer and Chemopreventive Effects

EVOO polyphenols exhibit notable anticancer properties. Oleuropein, hydroxytyrosol, and secoiridoids demonstrate significant antioxidative activity, inhibition of cell proliferation, induction of apoptosis, and modulation of inflammatory pathways linked to cancer development. In vitro and in vivo studies consistently report EVOO polyphenols’ chemopreventive effects across diverse cancer cell lines. Oleuropein specifically exhibits strong anticancer potential through radical-scavenging actions, metal-chelating activity, and inhibition of angiogenesis and platelet aggregation. Furthermore, polyphenolic constituents such as oleocanthal have shown promise as adjunctive therapeutic agents in cancer treatments given their potent anti-inflammatory and antioxidant capabilities [49,101,102,103,104,105,106].

#### 6.5.8. Gaps in Nutritional Research Related to EVOO

Despite extensive evidence of EVOO’s beneficial health effects, several critical gaps persist within the clinical and nutritional research domains. Most notably, robust, large-scale randomized controlled trials (RCTs) evaluating the long-term clinical impacts of EVOO consumption across diverse populations remain limited. There is an urgent need to define clearly optimal consumption levels, establish standardized phenolic profiles, and investigate how varying dietary patterns and individual genetic factors influence EVOO’s bioavailability and efficacy. Further research should aim to unravel the interactive mechanisms between EVOO constituents and other dietary components, providing a clearer, more definitive evidence base to support nutritional guidelines and public health recommendations concerning EVOO consumption.

## 7. Factors That Influence the Quality of Olive Oil

A high-quality EVOO has its origin in the orchard, with suitable cultivars, healthy olives, and ideal harvest times. Studies suggest that the highest phenolic content occurs in early harvests, in the first half the ripening/pigmentation stage, whereas overripe olives harvested in November or December will yield higher quantities of oil but with the lowest phenolic concentration and increased acidity [43,45,107].

**Figure 6:**
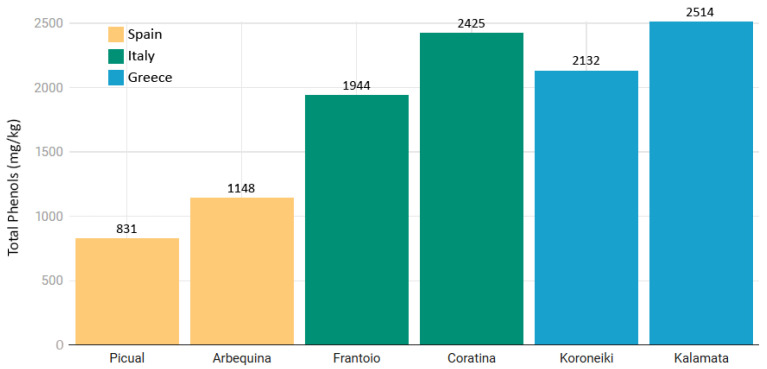
Variation in the average concentration of phenolic compounds (mg/kg) in monovarietal VOOs for 6 representative cultivars from Spain, Italy, and Greece [108,109,110].

It was reported that the concentration of total phenols differed by as much as 15-fold across 44 cultivars studied, though among the most common varieties from Spain, Italy, and Greece, as it can be observed in Figure 6, the differences were not so abrupt [108,109].

Free OO acidity refers to the percentage of free oleic acid found in OO [47]. Typically, the fatty acids in OO are esterified in the form of triglycerides, but free fatty acids are released when endogenous or exogenous lipases start breaking down these triglycerides [47]. When the olive fruit is intact, the oil is usually found in large vacuoles, separated from the watery part of the flesh that contains lipases [47]. These lipolytic enzymes can be found in the leaves as well [45] and also in fruit infested by the most frequent pest, the olive fruit fly (*Bactrocera oleae)* [43,47]. Oil produced from these infested olives has an increased acidity and a decimated phenolic content, up to a quarter of the original value [43]. Washing dirty or bruised olives for a prolonged time before milling is less preferable, as the additional water can serve as a medium for the dissolution of phenolic compounds, reducing their concentration in the extracted OO [45].

Dry summers stimulate earlier olive ripening and favor oil with higher phenol levels, but excessive temperatures induce an increase in the oil free acidity [43].

The ratios of fatty acids are variable with latitude and altitude and also with the use of fertilizers or irrigation. In colder environments, the PUFAs increase and SFAs decrease, while the oleic acid increases with altitude or the employment of ripening retardant fertilizers, and finally, both MUFAs and PUFAs are more abundant in crops growing in dryer environments [10]. Unexpected freezing temperature may initiate oxidative processes in the olives due to cell destruction, resulting in oil with a lower phenolic content [43].

Excessive nitrogen fertilization lowers the quality of the oil by reducing the phenol content, yet alpha-tocopherol content increases [43,45].

Most harvesting is performed through mechanical means (like trunk shakers), which produce significant damage to the fruit, more than 10 times when compared to hand-picking; therefore, it is essential that processing starts as soon as possible, as physical and chemical degradation increases exponentially after one hour, with the additional inconvenience of microorganism proliferation that will imprint undesirable organoleptic defects [43].

Technological extraction methods, process duration, and temperature are interconnected factors that influence the quality of the oil. If we compare methods, the observations might be surprising: even if the modern hammer crushing system induces a higher working temperature than a more traditional stone mill, the longer process required by the latter implies prolonged air exposure and therefore higher oxidation, so the stone-milled oil would have the lower content of total phenols of the two [45].

When the olive fruit is crushed, the oil vacuoles rupture and mix with the watery fraction of the olive fruit, initiating the lipolytic process. This process only stops once the oil is separated from the water and properly filtered, and this is why minimizing the processing time and the leaf content and having healthy and clean olive fruit is essential to maintain a low level of free acidity [43,45,47].

Unfiltered EVOO contains more polyphenols than filtered oil, especially those more polar that show increased affinity to the water droplets dispersed in the unfiltered oil [49]. Unfiltered EVOO may also offer superior flavor attributes, but it is important to note that most of these qualities originate in the fruit pulp suspension that also encourages fermentation and enzymatic activity, leading to the faster degradation of the unfiltered oil [43,45].

Destoning olives before crushing eliminates certain enzymes concentrated in the kernel, reducing phenolic oxidation and ultimately yielding oil with a higher phenolic content [45].

Intensive farming, increased irrigation, and selective cultivars offer better OO yields but not necessarily the highest quality in terms of health-enhancing attributes, yet growing consumer awareness of healthy foods and their willingness to pay more for them are driving demand for bioactive-rich cultivars [43].

## 8. EVOO Storage

EVOO and VOO have a better shelf life than seed oils, on the one hand because of the high oleic acid content and on the other hand because α-tocopherol, hydroxytyrosol, and secoiridoids act as synergic antioxidants [43,45,49]. A higher MUFA-to-PUFA ratio also improves the long-term oil oxidative stability [43].

Polar antioxidants tend to accumulate at the oil–air interface, where oxidation is more likely to occur, thus enhancing the protection against oxidative degradation by reducing oxygen permeability, acting as a physical barrier that slows oxidation [83]. This helps explain the polar paradox, where polar antioxidants sometimes outperform nonpolar ones in oil-based systems but only if certain concentrations are attained [83].

Storage conditions also influence EVOO’s polyphenol content, with studies showing that prolonged storage under diffused light, similar to supermarket levels, leads to the degradation of approximately 45% of total phenols within four months [111]. However, when stored in the dark, EVOO retains its antioxidant activity for up to eight months, and interestingly, hydroxytyrosol and tyrosol levels can increase during storage due to the hydrolysis of complex phenols, highlighting the dynamic nature of EVOO’s chemical composition over time [111].

Reducing the oxygen concentration in the bottle headspace to 2–5% significantly prolongs EVOO’s shelf life, particularly at cooler storage temperatures (10 °C). Low oxygen levels better preserve polyphenols, chlorophylls, and oil stability indicators, especially when dark glass packaging is used. Maintaining a low-oxygen headspace thus emerges as a crucial, yet often overlooked, packaging parameter. In comparison, EVOO stored at higher oxygen concentrations (10–21%) showed accelerated degradation, especially when combined with higher storage temperatures (28 °C). These findings emphasize the importance of controlled atmospheric packaging for premium oils, suggesting that even standard glass packaging can be optimized by simply adjusting the headspace composition, preferably with inert gases, to better preserve the oil’s chemical and sensory profile over time [112].

Packaging materials and storage temperatures critically influence the rate of EVOO degradation. Although tin packaging provides better light protection than glass, there are studies that indicated that only at low temperatures (6 °C) did both glass and tin containers help maintain EVOO quality, while higher temperatures (26 °C), particularly in tins, accelerated oxidative degradation and rancidity. Therefore, dark glass and cool temperatures are preferred in order to better preserve both chemical integrity and desirable sensory notes of EVOO, like bitterness and pungency. These findings underscore the delicate balance between packaging, temperature, and product longevity, demonstrating that storage temperature often plays a more decisive role than container type alone [113].

Other research showed that an average of 4 °C proved optimal for EVOO preserving, and even though −18 °C proved to show increased protective effects, it is also much more costly and impractical for long-term storage and transport [114].

Adding modified polysaccharides from *Lycium barbarum* to EVOO markedly improves oxidative stability under accelerated aging [115]. While technically effective, this practice raises regulatory and definitional concerns: EVOO must remain a pure product of the *Olea europaea* fruit. Introducing components from other plants, even if natural and beneficial, violates the legal and commercial standards defining EVOO, and such oils can no longer be marketed under this designation.

Proper home storage, including glass or tin-coated steel containers in cool, dark places, preserves quality. Most consumers (76%) in one study stored OO in closed cabinets, favoring tinplate containers (36.6%). However, more than a third of respondents stored OO in clear bottles and 16.4% in plastic, increasing degradation risks [8].

A study which evaluated over three years the stability of EVOO kept in unopened bottles under different temperature conditions revealed that the phenolic compounds experienced the most significant degradation, while tocopherols, squalene, and sterols showed only slight reductions, and fatty acids remained largely stable. Opening the bottle mid-way accelerated the degradation. At the end of the three-year period, only the high-phenol OO retained its organoleptic properties [114].

In conclusion, while technical innovations like headspace oxygen reduction, temperature regulation, and careful material selection can greatly improve EVOO shelf life within regulatory bounds, the use of additives from other botanical sources is incompatible with the fundamental definition of EVOO.

## 9. Effects of Cooking on EVOO

Lebanese, Greek, and Italian preferences for domestically produced oil reflects a Mediterranean trend valuing freshness and quality, with nearly half of participants from a recent study preferring to consume OO raw rather than use it for cooking [8].

EVOO and VOO are obviously best consumed raw to preserve their quality, but they are often exposed to heat in cooking. Heating affects not only their fatty acid composition but also their minor bioactive compounds. Research highlights concerns about the loss of beneficial substances and the formation of potentially harmful compounds, such as oxidized fatty acids and polymerized triglycerides [116].

The oxidation of EVOO’s minor compounds, particularly phenolic compounds like hydroxytyrosol, varies depending on temperature and cooking duration [117]. While some compounds degrade significantly, others, such as lignans and squalene, remain relatively stable [117]. Cooking techniques also influence EVOO’s oxidative stability—pan-frying leads to more degradation than deep-frying due to increased oxygen exposure, and microwave cooking accelerates the breakdown of antioxidants [117,118]. Yet, some studies reported that microwave cooking does not degrade OO; however, we disagree with these conclusions, as the exposure time in these studies was insufficient, and the OO samples, having minimal water content, would require a longer heating period to reach comparable thermal conditions to regular foods [117,118]. When boiling, phenolic compounds migrate into the water and degrade. Despite these changes, EVOO’s antioxidant profile helps protect fatty acids and vitamins from oxidation better than other oils [117].

Thermal treatments further accelerate the degradation, with frying, in particular, causing a sharp decline in hydroxytyrosol levels, with up to a 50% loss after just 10 min at 180 °C and less than 10% remaining after six frying cycles [111].

Cooking methods impact OO differently. Frying, especially repeated deep-frying, leads to oxidation, hydrolysis, and polymerization, potentially degrading bioactive compounds, despite OO’s resistance to thermal oxidation when compared to other frying oils. Boiling has a variable effect—while total phenolics remain stable at neutral pH, acidic conditions and the presence of certain vegetables (rich in metals like iron and copper) accelerate polyphenol loss through hydrolysis and leaching. To minimize degradation, OO should be added toward the end of the cooking process. Microwave heating is particularly damaging due to uncontrolled high temperatures, significantly degrading alpha-tocopherol and other beneficial compounds [116].

From a nutritional standpoint, consuming fried foods should be limited to occasional use due to the risk of oil absorption, which can increase calorie intake. Furthermore, when oils are reheated, they may generate potentially toxic degradation products. Therefore, it is crucial to use high-quality, stable frying oils and optimal frying conditions to ensure both the safety and the sensory quality of fried foods [65].

The type of oil used for frying varies by region and culinary tradition. In Europe, for instance, sunflower oil is more common in the east, OO in the Mediterranean, and rapeseed oil in the north [65]. Oils rich in MUFAs, such as OO, are actually considered more stable for frying due to their higher resistance to oxidation compared to oils with >3% PUFA, like sunflower or soybean oil [65,116]. While SFAs provide greater oxidative stability, they are less desirable nutritionally due to their links to cardiovascular diseases. OO is recommended for frying because of its superior stability at high temperatures and its beneficial fatty acid composition [65]. Studies performed during a 5-day-long process of oil reheating at 190 °C showed that OO degraded the slowest: it took 33 h for OO, 17 h for sunflower oil, and 4 h for linseed oil to reach the legal limit of Total Polar Compounds, and at the same time, the process generated the least amount of aldehides for OO [65,119].

While high temperatures during deep-frying alter the fatty acid composition, leading to increased saturated fatty acids and TFAs, EVOO remains more resistant to peroxidation than polyunsaturated-rich oils, reducing the formation of harmful lipid oxidation products (LOPs) [61].

Therefore, EVOO is considered a premium frying oil, offering both health benefits and better stability during storage and frying [65].

During cooking, EVOO undergoes chemical transformations due to heat and oxygen exposure, impacting both its major and minor components. Unlike other vegetable oils, EVOO remains stable at high temperatures due to its fatty acid profile, rich in monounsaturated fats and phenolic compounds. Although previously considered unsuitable for frying due to its relatively low smoke point, recent studies show that smoke point is not a reliable indicator of oil stability. EVOO outperforms other oils in resisting oxidation and producing fewer harmful byproducts, making it one of the best options for cooking [117].

EVOO can be used as the fat base in ice cream, offering a healthier and palatable alternative to traditional dairy fats [9]. A high polyphenol content enhances bitterness and pungency, potentially reducing consumer tolerance, yet the interaction of small-molecule polyphenols with milk proteins in food products like ice cream can influence the organoleptic properties, which appears to mask the bitterness [9]. Moreover, in vitro simulated digestion revealed that these interactions facilitated polyphenol release, potentially increasing antioxidant protection [9].

Cooking with EVOO usually results in beneficial interactions with food, enhancing the stability and bioavailability of certain bioactive compounds. For example, phenolic compounds migrate into foods like vegetables, improving their antioxidant content. The use of EVOO in tomato-based dishes increases the extraction of beneficial compounds from the tomatoes, enriching the final product. However, interactions between EVOO’s minor compounds and food macromolecules like proteins can alter nutrient absorption. While EVOO’s health benefits are well-documented, further research is needed to understand the new compounds formed during cooking and their impact on health [117].

## 10. Conclusions

An ever-growing body of research has consistently linked adherence to a Mediterranean diet, rich in EVOO, with lowered mortality and increased longevity. To fully understand the diet’s effects, it is necessary to further focus on the interplay between dietary intake and fasting periods, which are common on the Mediterranean region, which may synergistically influence metabolic health and disease risk [98,99,120,121].

The combination of intermittent fasting and EVOO intake may exert synergistic effects on human health through complementary and intersecting mechanisms. Intermittent fasting promotes metabolic flexibility, enhances insulin sensitivity, stimulates autophagy, and reduces systemic inflammation. Concurrently, EVOO, rich in monounsaturated fatty acids and bioactive polyphenols, contributes anti-inflammatory, antioxidant, and lipid-lowering effects. When used together, these strategies may amplify each other’s impact by modulating shared cellular signaling pathways, including AMPK activation, NF-κB inhibition, and improved mitochondrial function. This synergy may lead to more pronounced benefits in the prevention and management of metabolic syndrome, cardiovascular disease, and neurodegenerative disorders compared to either intervention alone.

Consumers represent the final link in the OO chain. Their ability to identify and select high-quality EVOO and also use it properly is crucial for maximizing potential health benefits. However, consumer choices could be influenced by price, biases, misconceptions, or insufficient knowledge, which could lead to suboptimal selection of EVOO products.

These findings highlight the need for future strategies to be developed towards enhancing consumer education and promoting informed choices in order to maximize EVOO’s nutritional and sensory benefits.

## Figures and Tables

**Table 1:** EFSA and WHO health claims and recommendations related to olive oil [53,54,55].

Health Claim/Recommendation	Conditions	Organization
Protection of LDL particles from oxidative damage	At least 5 mg hydroxytyrosol per 20 g olive oil; daily intake of 20 g	EFSA
Ensuring normal blood LDL cholesterol levels	Valid for foods high in unsaturated fats; oleic acid supports normal cholesterol levels	EFSA
Dietary fat intake recommendations	Total fat represents <30% of total energy intake; unsaturated fats preferred	WHO
Trans-fat intake recommendations	Trans-fats represent <1% of total energy intake (<2.2 g/day for 2000 kcal diet)	WHO

EFSA—European Food Safety Authority; LDL—low-density lipoproteins; WHO—World Health Organization.

**Table 2:** EVOO constituents with average proportions [10,43,46,47,49,65].

**Major fraction 98–99%**	saponifiable fraction mostly fatty acids in the form of TAGs, mainly triolein	≈75% MUFAs	55–83% ω-9 oleic acid <3.5% ω-7 palmitoleic acid <0.5% gadoleic acid, heptadecenoic acid	
		<25% PUFAs	3.5–21% ω-6 linoleic acid <1.5% ω-3 alpha-linolenic acid	
		<25% SFAs	7.5–20% palmitic acid <5% stearic acid <1% lignoceric acid, arachidic acid <0.5% heptadecanoic acid, behenic acid <0.1% myristic acid	
**Minor fraction** **1–2%**	unsaponifiable fraction (nonpolar)	hydrocarbons	squalene (2–9 g/kg), β-carotene	EVOO has 20–30% more squalene compared to VOO.
		tocopherols (lipophilic phenols)	10–350 mg/kg	In refined OO the tocopherols are lost. Alpha-tocopherol can be added.
		triterpenic alcohols and dialcohols		
		phytosterols	1–2.5 g/kg	But no cholesterol.
		pigments	chlorophylls, pheophitins	
	hydrophilic fraction (polar)	phenolic compounds 120–600 mg/kg (1–3% of pulp)	secoiridoids 90% (almost exclusive to *Olearaceae*)	Oleuropein, oleacin, oleocanthal, ligstrozide.
			phenolic acids	Benzoic and cinnamic acids derivatives.
			phenolic alcohols	Hydroxytyrosol tyrosol.
			lignans	Pinoresinol.
			flavonoids	Apigenin, luteolin.
			hydroxy-isochromans	
		volatile components	aldehydes, ketones and alcohols	

EVOO—extra-virgin olive oil; MUFAs—monounsaturated fatty acids; OO—olive oil; PUFAs—polyunsaturated fatty acids; SFAs—saturated fatty acids; TAGs—triacyl-glycerides; VOO—virgin olive oil.

## Data Availability

The original contributions presented in this study are included in the article. Further inquiries can be directed at the corresponding author.

## Abbreviations

The following abbreviations are used in this manuscript:

Aβ	amyloid-beta
AMPK	AMP-activated protein kinase
AP-1	activator protein 1
BCE	before current era
COX	cyclooxygenase
DNA	deoxyribonucleic acid
EFSA	European Food Safety Authority
EU	European Union
EVOO	extra-virgin olive oil
FAAH	fatty acid amide hydrolase
FDA	Food and Drug Administration
HDL	high-density lipoprotein
iNOS	inducible nitric oxide synthase
IOC	International Olive Council
Keap1	Kelch-like ECH-associated protein 1
L-DOPA	L-3,4-dihydroxyphenylalanine
LDL	low-density lipoprotein
LOPs	lipid oxidation products
LOX	lipoxygenase
MAO	monoamine oxidase
MD	Mediterranean diet
mTOR	mammalian target of rapamycin
MUFAs	monounsaturated fatty acids
NF-kB	nuclear factor kappa-light-chain-enhancer of activated B cells
Nrf2/ARE	nuclear factor erythroid 2-related factor 2/antioxidant response element
NSAID	nonsteroidal anti-inflammatory drug
OLC	oleocanthal
OO	olive oil
PUFAs	polyunsaturated fatty acids
RNS	reactive nitrogen species
ROS	reactive oxygen species
SIRT	sirtuins
SFAs	saturated fatty acids
TFAs	trans-fatty acids
VLDL	very-low-density lipoprotein
VOO	virgin olive oil
WHO	World Health Organization

## Appendix A

### Methodology

This review was conducted as a narrative synthesis of the peer-reviewed literature focused on the health effects of olive oil consumption, particularly in combination with intermittent fasting and within the broader framework of the Mediterranean diet. The aim was to integrate findings across clinical, preclinical, and in vitro research to explore the interconnected roles of olive oil components in modulating oxidative stress, inflammation, insulin resistance, and cognitive health.

To identify the relevant literature, comprehensive searches were carried out across ScienceDirect, PubMed and Google Scholar. A combination of keywords and Boolean operators was used to maximize sensitivity and relevance. Key terms included: olive oil, extra-virgin olive oil, EVOO, polyphenols, fasting, intermittent fasting, Mediterranean diet, oxidative stress, inflammation, insulin resistance, and cognitive function. In addition, specific articles were retrieved directly via DOI lookups from bibliographies encountered in primary and secondary sources.

Inclusion criteria encompassed human clinical trials, preclinical animal studies, in vitro investigations, reviews and official reports or position statements from authoritative bodies such as the WHO and the International Olive Council (IOC). To ensure thematic relevance, only studies that directly examined the effects of olive oil and/or fasting on metabolic, inflammatory, oxidative, or neurological endpoints were considered. Exclusion criteria comprised non-original research (e.g., commentaries, news articles), papers not isolating the effects of olive oil or fasting (e.g., multi-component interventions without clear attribution) and publications not available in English. No restrictions were applied based on study location or sample demographics.

A total of approximately 300 sources were initially retrieved, including journal articles, book chapters, books, and official documents. After screening for relevance and methodological quality, only 122 sources were selected and cited in the final manuscript. While the majority of references are from the last decade, a limited number of older but seminal works were also included to provide historical or mechanistic context.
